# Functional Analysis of MADS-Box Gene Family in Stress Response and Prospects of Breeding Application

**DOI:** 10.3390/plants15081262

**Published:** 2026-04-20

**Authors:** Jiaxuan Wang, Hongying Wang, Mengyao Li, Yujie Chen, Bingyan Song, Yingying Li, Xuhui Meng, Jie Li, Wenting Lu, Yi Gao, Yao Zhang, Aoxue Wang

**Affiliations:** 1 College of Life Sciences, Northeast Agricultural University, Harbin 150030, China; ngx1007372074@163.com (J.W.); wanghongying2024@163.com (H.W.); 19913405928@163.com (M.L.); 2020211521@jsnu.edu.cn (Y.C.); n020107@126.com (B.S.); yizhixiaopangying@163.com (Y.L.); 18865786885@163.com (X.M.); 19819729076@163.com (J.L.); 15145201366@163.com (W.L.); 15046084962@163.com (Y.G.); 2 College of Horticulture and Landscape Architecture, Northeast Agricultural University, Harbin 150030, China

**Keywords:** MADS-box transcription factors, stress, molecular breeding

## Abstract

The MADS-box family is a multifunctional family of transcription factors characterized by the presence of a unique MADS domain, which plays an important part in regulating essential biological processes, including metabolic synthesis and the stress response. In this review, we analyze the structural features and classification of MADS-box proteins, then summarize the functions of the MADS-box family in the stress response. The MADS-box family can directly regulate downstream functional genes by binding to the CArG-box in the promoters of target genes, thereby influencing growth, development, and stress responses. Also, MADS-box transcription factors can form protein complexes with both MADS-box proteins and other types of transcription factors and chromatin regulatory proteins to modulate the chromatin state or transcriptional activation. Furthermore, they can regulate plant physiological responses by facilitating the synthesis of essential signaling molecules, including hormones and non-coding RNA. Finally, we discuss the potential of the MADS-box family in crop molecular breeding, offering a novel approach for developing high-yield and stress-resistant cultivars for solving global food security and climate change challenges.

## 1. Introduction

The MADS-box family constitutes a critical group of transcription factors in plants, governing diverse fundamental biological processes such as growth, development, metabolic regulation, and stress responses. The designation “MADS” is an acronym derived from the four founding members: MCM1 from *Saccharomyces cerevisiae*, AGAMOUS (AG) from *Arabidopsis thaliana*, DEFICIENS (DEF) from *Antirrhinum majus*, and SRF from *Homo sapiens* [1].

MADS-box genes play pivotal regulatory roles throughout the plant life cycle, encompassing seed germination, root and shoot morphogenesis, apical meristem maintenance, leaf development, and fruit ripening [2]. Notably, this family is instrumental in the evolution of floral morphology and the regulation of floral organ identity. By controlling flowering time and floral architecture, MADS-box genes directly determine fruit development and yield potential [3,4,5]. Consequently, precise manipulation of these genes offers a strategy to optimize crop growth cycles and enhance adaptation to climate change.

Furthermore, the MADS-box family is integral to stress response networks, conferring tolerance to abiotic stresses such as drought, salinity, extreme temperatures, and ion toxicity, as well as resistance to biotic stresses like fungal pathogens [6]. By integrating and modulating the expression of downstream stress-responsive genes, MADS-box proteins enhance crop survival and resilience under adverse environmental conditions.

This review elucidates the structural characteristics, classification, and evolutionary history of MADS-box proteins. We systematically summarize their functional roles in plant metabolism and stress responses, and discuss their potential applications in molecular breeding. Ultimately, this work aims to provide a comprehensive reference for understanding MADS-box regulatory networks and to offer novel insights for agricultural genetic improvement.

## 2. The Structure and Classification of the MADS-Box Proteins

Based on sequence homology within the MADS-domain, these transcription factors are classified into Type I and Type II. Type I factors, designated as M-type, encode shorter proteins characterized by a conserved region limited to the MADS-domain. In contrast, Type II factors, known as MIKC-type, possess a more extended architecture comprising the MADS-domain in conjunction with additional conserved regions, notably the K-domain. Type II factors play pivotal roles in plant growth, reproductive development, and stress responses [7,8,9]. Phylogenetically, Type I proteins are divided into four subgroups: Mα, Mβ, Mγ, and Mδ. Type II factors comprise two subfamilies: MIKCc and MIKC* [10,11]. In *Arabidopsis thaliana*, members of the MADS-box family exhibit a broad yet distinct expression landscape. Transcriptomic analyses reveal that out of the 107 identified MADS-box genes, 101 are expressed in at least one of four major tissues: roots, rosette leaves, inflorescences, or siliques [11]. Beyond this widespread presence, the family displays marked spatial specificity [11]. Type II members are predominantly associated with reproductive development—governing floral organ identity, flowering time, and fruit formation—and consequently show highly restricted expression in reproductive organs. In contrast, Type I members display diverse expression patterns [11]. Specific subclades within Type I are localized to distinct vegetative or reproductive tissues: certain Mα (e.g., *AGL56*) and Mβ (e.g., *AGL26*) subfamily members are primarily root-expressed, whereas others, such as the Mα member *AGL85*, are leaf-specific. Furthermore, distinct expression in inflorescences or siliques is observed in members of the Mγ (e.g., *AGL36*, *AGL90*) and Mδ (e.g., *AGL104*) subfamilies [11]. Type II MADS-box transcription factors are primarily divided into two structural groups: MIKCc and MIKC*. Type II MADS-box transcription factors are further classified into distinct subfamilies, including AGAMOUS (AG), APETALA1 (AP1), FRUITFULL (FUL), SEPALLATA (SEP), APETALA3/PISTILLATA (AP3/PI), SHORT VEGETATIVE PHASE (SVP), SUPPRESSOR OF OVEREXPRESSION OF CONSTANS 1 (SOC1), TM3, FLOWERING LOCUS C (FLC), AGL6, and AGL17. The functional roles of these subfamilies have been partially elucidated [12]. The AGL17 subfamily predominantly regulates root development, with expression localized to the root apical meristem, lateral root primordia, and vascular tissues. It governs root system architecture and facilitates the transition from vegetative to reproductive growth [13,14]. The SOC1 and SVP subfamilies function as floral promoters and repressors, respectively. They integrate exogenous cues, such as ambient temperature, with endogenous hormonal signals to modulate the expression of downstream targets, thereby orchestrating the flowering process. Notably, certain SOC1 subfamily members, including *AGL42* in Arabidopsis, also contribute to the regulation of root development [15,16]. The AP3/PI subfamily, which corresponds to B-function in the ABCDE model, plays a critical role in floral and fruit development. During floral organogenesis, these proteins interact with PI-homologous partners to specify petal and stamen identity. Functioning as E-class proteins, the SEP subfamily acts as a “molecular glue,” serving as an essential scaffold for the formation of functional tetrameric complexes, such as AP1-AP3-PI-SEP3 and AG-SEP3-AP3-PI [17,18,19,20]. Phylogenetically closely related to the SEP subfamily, the AGL6 subfamily represents an extension of E-class function, regulating floral organ identity and meristem determination [21].

MADS-box proteins are structurally characterized by a conserved MADS-domain accompanied by I, K, and C domains [22]. Located at the N-terminus, the MADS-domain is primarily responsible for binding to the promoters of downstream target genes. These target sequences predominantly consist of (CC(A/T)6GG) motifs and related variants, collectively termed CArG boxes. Following the MADS-domain, the I domain forms an α-helix that interacts with the MADS-domain α-helix, thereby facilitating dimerization [23,24]. Consequently, these factors facilitate the assembly of dimers and heterotetrameric complexes [24]. In Arabidopsis, for instance, the floral homeotic proteins AP3 and PI first form a heterodimer, which subsequently associates with AP1 and SEP3 to constitute a tetrameric complex that specifies petal cell identity [24]. Composed of hydrophobic α-helices, the K domain is critical for protein dimerization and oligomerization, which modulates transcriptional regulatory functions [5]. Furthermore, the K domain mediates interactions with other transcriptional regulators, enabling the assembly of complexes that precisely control downstream gene expression [25]. The C-terminal domain modulates transcriptional activity by mediating interactions with other transcription factors or co-regulators [26].

## 3. The Function of the MADS-Box Gene Family in Stress Response

### 3.1. Response of the MADS-Box Gene Family to Heat Stress

#### 3.1.1. Functional Diversity of MADS-Box Genes in Heat Stress Responses

**Figure 1:**
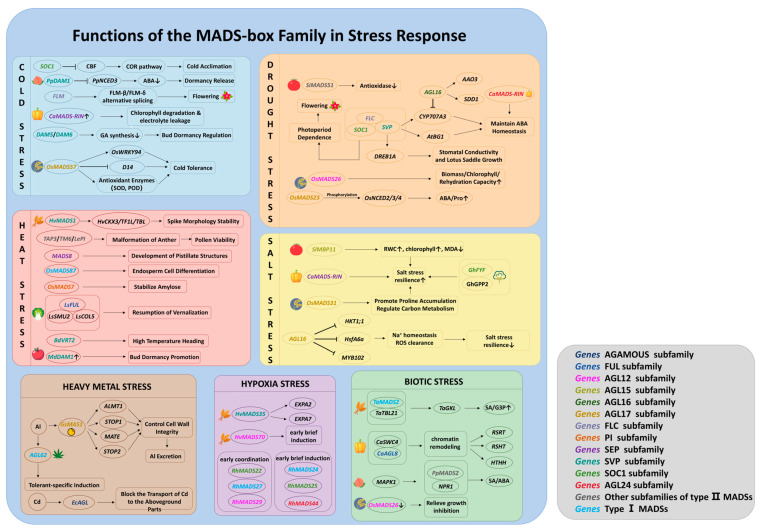
Mechanism of the MADS-box gene family in stress response. This schematic illustrates the complex regulatory network where MADS-box transcription factors act as central hubs integrating various upstream signals to modulate downstream physiological processes and stress responses. Upward and downward arrows denote upregulation and downregulation, respectively.

The MADS-box gene family plays a pivotal role in plant responses to heat stress, modulating critical biological processes including reproductive development, morphogenesis, seed development, growth cycle regulation, and energy metabolism. For instance, *MADS8* (SEP subfamily) governs floral meristem determination and ovule formation in barley and rice under high-temperature conditions, facilitating proper pistil development [27]. In barley, *HvMADS1* (SVP subfamily) stabilizes panicle morphology under heat stress by binding to floral differentiation genes, such as *HvCKX3*, *HvTFL1L*, and *HvTB1L*, thereby regulating floral differentiation and hormone signaling to prevent heat-induced structural anomalies (Figure 1) [28]. Conversely, in tomato, heat stress suppresses the expression of class B MADS-box genes (*TAP3*/*TM6*/*LePI*), resulting in the homeotic conversion of anthers to carpellary structures and reduced pollen viability, ultimately compromising fertility (Figure 1) [29]. RNA silencing of *TM6* results in anther malformation and reduced pollen viability under heat stress, confirming its critical role in maintaining floral organ stability [30]. Regarding developmental timing, the MADS-box transcription factor BdVRT2 (SVP subfamily) in the temperate grass model *Brachypodium distachyon* facilitates the resumption of vernalization following heat interruption and modulates vernalization duration (Figure 1) [31]. In rice, short-term heat stress (48 h) upregulates type I MADS genes (e.g., *OsMADS87*), exacerbating grain heat sensitivity. Furthermore, suppression of *OsMADS7* (AGL17 subfamily) in rice endosperm under heat stress stabilizes amylose content and preserves panicle fertility (Figure 1) [32]. Rice *OsMADS87* influences seed size and vigor under high temperatures by regulating the endosperm cellularization process. Notably, overexpression of *OsMADS87* alleviates the reduction in seed size typically induced by heat stress (Figure 1) [30]. Conversely, deletion of these genes enhances seed heat tolerance and increases seed size; although this negatively affects endosperm cellularization, it ultimately mitigates heat-induced grain damage [33]. Additionally, in *Lactuca sativa* L., the LsFUL (AGL8/FUL subfamily) protein associates with LsSMU2 and LsCOL5 to exert synergistic regulatory effects on bolting time under high temperature (Figure 1) [34]. Finally, in apple, elevated temperatures induce *MdDAM1* (SVP subfamily) expression, which promotes bud dormancy and increases chilling requirements (Figure 1) [35].

#### 3.1.2. Current Knowledge Gaps in MADS-Box-Mediated Heat Stress Mechanisms

Despite these findings, the specific mechanisms underlying MADS-box-mediated heat stress responses remain insufficiently characterized, representing a significant knowledge gap. The current literature predominantly focuses on model systems and major cereals such as rice and barley, with limited investigation into staple crops like maize or horticultural species such as Chinese cabbage. Moreover, existing studies often rely on reductionist approaches examining single genes, traits, or pathways in isolation. There is a paucity of research regarding synergistic or antagonistic interactions among MADS-box genes within a species, cross-regulatory crosstalk with core heat stress pathways (e.g., HSF-HSP) and hormone signaling networks, and tissue-specific regulatory variations. Additionally, the spatiotemporal dynamics of heat stress are frequently overlooked; the differential effects of acute versus chronic heat exposure, as well as stress imposed at distinct developmental stages, on MADS-box function remain unclear. Crucially, most studies have established correlations between transcriptional changes and phenotypes without elucidating the intermediate physiological mechanisms, such as hormone metabolism or cell differentiation, leaving the “molecular–physiological–phenotypic” regulatory continuum incomplete.

#### 3.1.3. Future Research Directions for MADS-Box Genes in Thermal Adaptation

To address these limitations, future research should prioritize the following directions. First, species and gene coverage must be expanded by leveraging omics and transgenic technologies to validate core candidate genes in non-model crops. Second, comprehensive regulatory networks should be constructed to elucidate gene–gene interactions and their crosstalk with core stress pathways. Third, investigations should account for the spatiotemporal characteristics of heat stress and combined stress scenarios (e.g., heat co-occurring with biotic stress or drought), with functional validation conducted under field conditions. Ultimately, these efforts aim to facilitate the breeding of heat-tolerant cultivars or varieties with reduced chilling requirements adapted to warming winters, thereby mitigating the agricultural impacts of global climate change.

### 3.2. Response of the MADS-Box Gene Family to Cold Stress

#### 3.2.1. Regulation of the Core CBF Cold Response Pathway by MADS-Box Genes

The MADS-box transcription factor family is implicated in the regulation of plant responses to cold stress. Research indicates that cold stress significantly upregulates *CaMADS-RIN* (SEP subfamily) expression in pepper. Knockdown of CaMADS-RIN results in chlorophyll degradation, increased electrolyte leakage, and reduced cold tolerance; conversely, overexpression markedly enhances cold stress tolerance (Figure 1) [36]. The CBF-dependent pathway constitutes a fundamental regulatory network for cold adaptation, wherein MADS-box proteins can either activate or repress *CBF* expression. In Arabidopsis, the MADS-box transcription factor SOC1 (TM3/SOC1 subfamily) directly interacts with the CArG box in the CBF promoter, thereby diminishing *CBF* transcription levels and negatively modulating the cold response (Figure 1) [37]. In rice, *OsMADS57* (AGL17 subfamily) is induced by cold and improves tolerance by upregulating antioxidant enzymes (e.g., SOD, POD) and genes associated with photosynthetic protection (Figure 1) [38]. Also, rice *OsMADS57* activates *OsWRKY94* and represses *D14* in a temperature-dependent manner to enhance cold tolerance. Consequently, *OsMADS57* overexpression lines exhibit significantly higher survival rates at 4 °C compared to wild-type plants (Figure 1) [30]. In Arabidopsis, the *FLOWERING LOCUS M* (*FLM*, FLC subfamily) gene generates isoforms via alternative splicing that form heterodimers with *SVP* to modulate flowering time in response to temperature fluctuations (Figure 1) [30]. Furthermore, *SOC1* balances cold tolerance and floral development by suppressing the CBF/COR cold response pathway [30].

#### 3.2.2. Multidimensional Regulatory Mechanisms of MADS-Box Genes in Cold Adaptation

Beyond regulating the primary CBF-mediated signaling pathway, MADS-box transcription factors establish a multifaceted regulatory system by integrating signals from crucial hormones such as abscisic acid (ABA) and gibberellin (GA), facilitating adaptive responses to low temperatures. The MADS-box transcription factor *PpDAM1* (SVP subfamily) directly binds to the CArG motif in the promoter region of *PpNCED3*, a key rate-limiting gene in the ABA biosynthesis pathway. This interaction activates *PpNCED3* expression and enhances ABA levels, mediating ABA-dependent cold resistance (Figure 1) [39]. Additionally, MADS-box proteins regulate GA levels to indirectly modulate cold stress responses. *DAM* genes, belonging to the SVP/*StMADS11* evolutionary lineage, inhibit critical genes in the GA signaling pathway (e.g., GA biosynthesis genes), thereby reducing GA accumulation. During winter, sustained elevated expression of *DAM5* and *DAM6* maintains bud dormancy by suppressing GA synthesis or enhancing GA inactivation, preventing premature germination under low-temperature conditions (Figure 1) [40]. At the physiological level, the MADS-box family enhances cold resistance via a dual mechanism: safeguarding the structure and function of the photosynthetic system to ensure stable energy metabolism, and activating the antioxidant defense system to mitigate ROS-mediated oxidative damage [30,41]. In summary, the MADS-box transcription factor family exhibits functional diversity and intricate regulatory networks, establishing a plant cold stress response system through three dimensions: CBF-dependent pathway modulation, hormone signal integration, and targeted physiological protection.

### 3.3. Response of the MADS-Box Gene Family to Drought Stress

#### 3.3.1. Positive Regulatory Roles of MADS-Box Genes in Drought Stress Responses

The MADS-box gene family plays a pivotal role in regulating plant responses to drought stress, with underlying mechanisms extensively characterized. Central to this regulation is the abscisic acid (ABA) signaling pathway. ABA induces stomatal closure, thereby attenuating plant respiration and transpiration [42]. As carotenoids serve as the direct precursors of ABA, MADS-box transcription factors exert regulatory control over nearly all stages of carotenoid metabolism, ranging from biosynthesis to degradation [43,44]. Specifically, the oxidative cleavage of C40 carotenoids into C15 xanthophylls, catalyzed by 9-cis-epoxycarotenoid dioxygenase (NCED), represents the first committed and irreversible step in ABA biosynthesis. MADS-box transcription factors modulate *NCED* expression by directly binding to CArG-box motifs within the promoters of NCED genes [39,43]. In rice, *OsMADS23* (AGL17 subfamily) is phosphorylated by *SAPK9*, facilitating the accumulation of endogenous ABA and proline by modulating critical biosynthetic genes (*OsNCED2*/*3*/*4* and *OsP5CR*), thereby markedly improving tolerance to drought and salinity (Figure 1) [45]. Similarly, the pepper *CaMADS-RIN* gene is activated by diverse abiotic stimuli, including drought, contributing to osmotic stress responses via the ABA signaling pathway (Figure 1) [36]. In Arabidopsis, the *SVP* gene maintains ABA homeostasis and enhances drought resistance by downregulating ABA catabolic genes (*CYP707A1*/*3*) and upregulating the ABA activation gene *AtBG1* (Figure 1) [30]. Concurrently, *SVP* modulates early drought-responsive genes such as *DREB1A*, thereby influencing stomatal conductance and rosette growth (Figure 1) [30]. Rice *OsMADS26* (AGL12 subfamily) functions as a negative regulator; RNA silencing of this gene significantly improves biomass, chlorophyll content, and recovery capacity upon rewatering under drought conditions (Figure 1) [30]. However, the regulatory role of *OsMADS26* remains debated, as some studies suggest it may positively regulate reactive oxygen species (ROS)-related genes [30]. Regarding developmental plasticity, the Arabidopsis *SOC1*, *FLC*, and *SVP* genes constitute a regulatory module that controls drought-escape flowering via a photoperiod-dependent mechanism (Figure 1) [30]. Specifically, *SOC1* induces flowering under long-day conditions, whereas the *FLC*/*SVP* complex suppresses flowering under short-day conditions, ensuring life cycle completion in favorable environments (Figure 1) [30].

#### 3.3.2. Negative Regulatory Roles of MADS-Box Genes in Drought Stress Responses

Conversely, certain MADS-box factors function as negative regulators. In *Arabidopsis*, *AGL16* (AGL17 subfamily) modulates the expression of ABA-metabolizing genes (*CYP707A3*, *AAO3*) and the stomatal density-regulating gene *SDD1* by binding to CArG motifs in their promoter regions, negatively controlling drought resistance through stomatal density and ABA metabolism (Figure 1) [46]. Whole-genome analysis in Foxtail Millet (*Setaria italica*) identified 25 drought-responsive *SiMADS* genes, with *SiMADS51* (a member of MIKC-type MADS) serving as a prominent negative regulator; its overexpression increases malondialdehyde levels and reduces antioxidant enzyme activity, substantially compromising drought resistance (Figure 1) [47].

#### 3.3.3. Conserved and Species-Specific Regulatory Features of MADS-Box Genes in Drought Adaptation

Collectively, MADS-box genes regulate drought stress through diverse pathways, including ABA signaling, stomatal development, and the biosynthesis of osmotic regulatory substances. Recent studies indicate that these genes exhibit bidirectional regulatory functions, with functional differentiation intricately linked to species evolution and ecological adaptability. While the ABA pathway represents a conserved mechanism—as evidenced by *OsMADS23* (AGL17 subfamily) triggering ABA synthesis [45], *AGL16* regulating ABA metabolism [46], and *CaMADS-RIN* responding to ABA signals [36]—species-specificity is markedly evident. For instance, although both *SiMADS51* in monocotyledonous crops [47] and *AGL16* in dicotyledonous plants [46] serve as negative regulators, they operate through distinct downstream target genes. Furthermore, response patterns of MIKC-type MADS-box genes in *Camelina sativa* [48] and *LcMADS* genes in lychee (*Litchi chinensis*) [49] exhibit significant differences, correlating with distinct drought resistance strategies such as tolerance in cereals versus long-term stress adaptation in woody species. Subsequent research should clarify the regulatory networks of MADS-box genes across various plant species to identify additional molecular targets for enhancing drought resistance in crops.

### 3.4. Response of the MADS-Box Gene Family to Salt Stress

#### 3.4.1. Differential Expression Profiles of MADS-Box Genes Under Salt Stress

Salt stress represents a major environmental constraint that limits plant growth and productivity by inhibiting seed germination and impairing development, flowering, and fruiting [50,51,52]. Emerging evidence underscores the critical role of MADS-box proteins in mitigating salt stress. Genomic profiling in sugar beet (*Beta vulgaris*) identified several *BvMADS* members sensitive to salt stress [53]. Similarly, in banana (*Musa* spp.), the expression of *MbMADS27* (a member of the SOC1 subfamily) is downregulated under high-salinity conditions [54]. This repression is mirrored by other members of the same subfamily, including *MbMADS71*, *MbMADS77*, and an unnamed homolog. In contrast, *MbMADS76* (TM3/SOC1 subfamily) acts as a core upregulated gene within this subfamily under salt stress [54]. This functional divergence, characterized by concurrent activation and repression among TM3/SOC1 members, constitutes a pivotal regulatory mechanism for banana’s salt stress response. Furthermore, multiple Type II MIKCc genes from the AG, SEP/AGL2, and AGL17 subfamilies exhibit non-significant transcriptional fluctuations [54]. Together with the TM3/SOC1 subfamily, these genes form a comprehensive MADS-box regulatory network mediating banana’s adaptation to salinity stress [54].

#### 3.4.2. Bidirectional Regulatory Functions of MADS-Box Genes in Salt Tolerance

In rice, *OsMADS31* (AGL17 subfamily) enhances salt tolerance by stimulating antioxidant activity, promoting proline accumulation, and modulating carbon metabolism and hormone signaling (Figure 1) [55]. Conversely, in Arabidopsis, *AGL16* acts as a negative regulator; it binds to CArG motifs in the promoters of *HKT1;1*, *HsfA6a*, and *MYB102*, repressing their expression. This repression disrupts Na^+^ homeostasis and impairs ROS scavenging, ultimately reducing salt tolerance (Figure 1) [46]. In tomato, *SlMBP11* (AGL15 subfamily), is induced by salt stress. Overexpression of *SlMBP11* enhances salt tolerance by increasing the relative water content (RWC) and chlorophyll levels while mitigating oxidative damage, evidenced by reduced malondialdehyde accumulation and electrolyte leakage (Figure 1) [30]. In contrast, its homolog, *SlMBP8* (AGL15 subfamily), acts as a negative regulator, with RNAi lines exhibiting improved tolerance to both salt and drought stresses [30]. Heterologous overexpression of cotton *GhFYF* (SOC1 subfamily) in *Arabidopsis* significantly improves salt tolerance, mediated by its interaction with the stress-related protein GhGPP2 (Figure 1) [56]. In pepper, *CaMADS-RIN* functions as a positive regulator by activating ROS scavenging mechanisms, enhancing osmolyte synthesis, and modulating downstream stress response genes, thereby maintaining cellular redox and osmotic homeostasis (Figure 1) [36].

#### 3.4.3. Unresolved Mechanisms and Current Knowledge Gaps in Salt Stress Regulation

Collectively, these findings underscore the functional diversity of MADS-box transcription factors in salinity responses. Although their involvement is well-documented across species, the underlying regulatory networks remain incompletely characterized. In particular, the direct downstream targets and specialized regulatory mechanisms governing specific members, such as *MbMADS27*, warrant further investigation.

### 3.5. Response of the MADS-Box Gene Family to Heavy Metal Stress

#### 3.5.1. Functional Characterization of MADS-Box Genes Under Heavy Metal Stress

Anthropogenic contamination has resulted in the persistent accumulation of phytotoxic heavy metals, such as cadmium (Cd), nickel (Ni), and aluminum (Al), in soil systems, posing severe threats to plant growth and ecosystem integrity. To mitigate heavy metal toxicity, plants have evolved sophisticated molecular defense mechanisms, within which MADS-box transcription factors serve as pivotal regulators of adaptive responses.

Investigations into MADS-box-mediated heavy metal stress responses have largely centered on aluminum (Al) toxicity. In soybean (*Glycine soja*), a member of MIKC-type MADS, *GsMAS1* (AGL17 subfamily) acts as a key regulator of Al tolerance by activating the expression of downstream genes, including *ALMT1*, *STOP1*, *MATE*, and *STOP2*, thereby enhancing resistance and maintaining cell wall integrity (Figure 1) [57,58]. Similarly, in flax (*Linum usitatissimum*), the transcription of *AGL62* (type I MADS gene) is specifically induced in Al-tolerant genotypes, suggesting a strong association with natural variation in Al tolerance (Figure 1) [59]. Beyond aluminum, MADS-box genes also modulate responses to other heavy metals. In *Erigeron canadensis*, *EcAGL* expression is significantly upregulated under Cd stress; heterologous expression in Arabidopsis revealed that this gene impedes Cd translocation to shoots without compromising root accumulation (Figure 1) [60].

#### 3.5.2. Research Limitations and Future Perspectives on Heavy Metal Stress Responses

Collectively, these findings establish the MADS-box family as an integral component of the plant heavy metal detoxification machinery, governing processes such as metal transport, stress signaling, and structural adaptation. However, notable limitations persist in the current literature. Existing studies are restricted to a narrow range of non-model plant species and have primarily examined Al, Ni, and Cd stress. Moreover, most reports rely predominantly on expression profiling without elucidating underlying molecular mechanisms or regulatory pathways. Future research should expand the scope to include additional heavy metals (e.g., chromium, lead, mercury) and diverse plant species. Crucially, efforts must focus on dissecting the specific signaling pathways and regulatory networks controlled by MADS-box factors to comprehensively understand their role in heavy metal stress resilience.

### 3.6. Response of the MADS-Box Gene Family to Hypoxia Stress

#### 3.6.1. Spatiotemporal Regulatory Patterns of MADS-Box Genes Under Hypoxia Stress

Waterlogging stress has emerged as a prevalent abiotic constraint, exacerbated by global climate change and increased precipitation patterns. Accumulating evidence indicates that MADS-box transcription factors serve as pivotal regulators of waterlogging tolerance across diverse plant species. These transcription factors exhibit temporally coordinated expression profiles, facilitating staged adaptive responses to hypoxic environments. Conserved expression motifs were observed in barley (*Hordeum vulgare*), where *HvMADS35* and *HvMADS70* (SVP and AGL12 subfamilies, respectively) constitute a core regulatory module for waterlogging response. *HvMADS35* was specifically induced in root tissues and is predicted to physically interact with the expansins EXPA2 and EXPA7, potentially facilitating cell wall remodeling during prolonged hypoxia. In contrast, *HvMADS70* displayed transient early induction but was downregulated after 10 days, implicating it in initial hypoxic signal transduction (Figure 1) [61]. In *Rhododendron hainanense*, distinct temporal expression peaks were observed under hypoxic conditions: *RhMADS24*, *RhMADS25*, and *RhMADS44* (type I, SOC1, and AGL24 clades) reached peak expression at 3, 12, and 24 h, respectively. This temporal divergence suggests stage-specific functions during hypoxia stress. Conversely, *RhMADS22*, *RhMADS27*, and *RhMADS29* (SOC1, type I, and AGL12 clades) formed a co-regulatory module, showing synchronous upregulation at 3 h, which implies a collaborative role in early stress signaling cascades (Figure 1) [62].

#### 3.6.2. Current Limitations and Future Directions in Hypoxia Stress Regulation

Collectively, these findings substantiate that MADS-box transcription factors coordinate hypoxic adaptation through stage-specific functional specialization. Their co-expression patterns provide a theoretical framework for understanding plant adaptation to fluctuating oxygen levels and offer candidate targets for breeding waterlogging-resistant crops. Nevertheless, significant gaps persist in the current literature. Research on major cereal crops (e.g., rice, wheat) and vegetables (e.g., cabbage, tomato) remains limited, particularly regarding functional validation via heterologous expression. Furthermore, the underlying molecular mechanisms of identified genes remain largely elusive. Future research should leverage multi-omics approaches to systematically identify hypoxia-responsive MADS-box genes, elucidate their specific regulatory mechanisms, and utilize gene editing technologies to validate their functions for crop improvement.

### 3.7. Response of the MADS-Box Gene Family to Biotic Stress

#### 3.7.1. Functional Diversity of MADS-Box Genes in Biotic Stress Defense

MADS-box transcription factors mediate disease resistance across diverse plant species through multiple molecular pathways, including pathogen recognition, modulation of defense signaling, and protein–protein interactions. Certain members indirectly influence disease resistance by modulating pathogen virulence factors.

Genome-wide expression profiling reveals that wheat MIKC-type MADS-box genes are broadly upregulated under diverse biotic stresses, including Fusarium head blight, rice blast, stripe rust, powdery mildew, and wheat leaf spot. Among these, the floral homeotic subfamilies (AP1, AP3, PI, AG/STK, AGL6, and SEP) exhibit the highest transcript abundance in spikes challenged by *Fusarium graminearum*. Moreover, *AP1*-like genes display specific upregulation in response to *Magnaporthe oryzae* infection, suggesting their involvement in pathogen-specific defense mechanisms [63]. Conversely, some MADS-box genes act as negative regulators. Downregulation of *OsMADS26* in rice enhances resistance to *Magnaporthe oryzae* and *Xanthomonas oryzae* and improves drought tolerance without compromising growth, illustrating the decoupling of defense mechanisms from developmental processes (Figure 1) [64]. This offers a promising strategy for precise genetic improvement in crop breeding. In wheat, *TaMADS2* (type I MADS-box gene) interacts with *TaTBL21* to upregulate *TaGKL*, facilitating the accumulation of defense metabolites such as salicylic acid (SA) and glycerol-3-phosphate (G3P), thereby enhancing resistance to stripe rust (Figure 1) [65]. This elucidates the mechanism by which MADS-box factors regulate defense metabolite production.

#### 3.7.2. Molecular Regulatory Mechanisms of MADS-Box Genes in Biotic Stress Responses

MADS-box transcription factors also modulate chromatin states and transcriptional activation via protein interactions, thereby promoting downstream defense gene expression. In pepper, *CaAGL8* (FUL subfamily) interacts with *CaSWC4* to activate defense-related genes (e.g., *RSRT*, *RSHT*, *HTHH*) through chromatin remodeling, initiating the resistance response (Figure 1) [66]. In citrus, co-expression of a CsMIKC-type MADS-box factor with *PgSCP*, a protein secreted by the biocontrol yeast *Pichia galeiformis*, enhances transcriptional activation of PR1-like proteins and ATPase promoters, improving resistance to green mold and providing a novel strategy for integrating biocontrol with genetic regulation [67]. The *Prunus persica* MADS-box gene *PpMADS2* overexpressed in Arabidopsis interacts with *NPR1*, conferring DNA-binding capability essential for transcribing SA-dependent pathogenesis-related (PR) genes and ABA-induced CalS genes, thereby facilitating β-aminobutyric acid (BABA)-induced resistance (Figure 1) [68]. This process involves MAPK1-mediated post-translational modification of MADS2. Overexpression of *PpMADS2* (a member of MIKC-type MADS) significantly improves fungal resistance in transgenic Arabidopsis, underscoring the critical role of MADS-box factors in the crosstalk between hormone signaling and disease resistance mechanisms.

In conclusion, plant MADS-box transcription factors exhibit complex and multifaceted roles in disease resistance mechanisms. Comprehensive elucidation of their molecular mechanisms and translational potential is critical for breeding disease-resistant crops and implementing sustainable pest management strategies, thereby underscoring the substantial research significance and practical applications of this field.

## 4. Breeding Potential of the MADS-Box Gene Family

**Figure 2:**
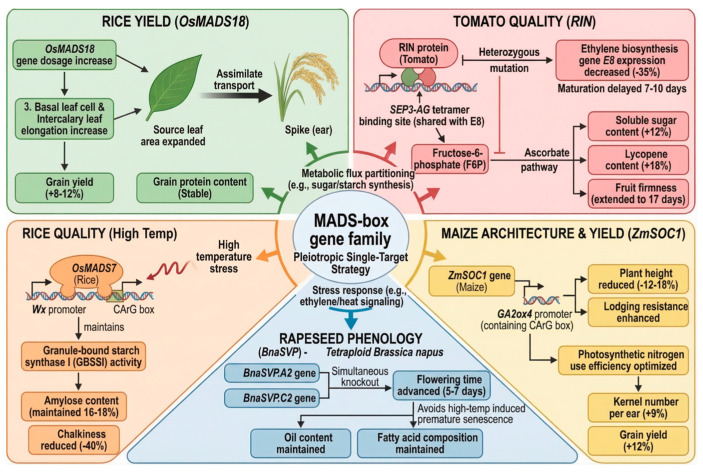
Multifaceted regulatory mechanisms of the MADS-box gene family in crop molecular breeding. Overview of key examples demonstrating yield improvement (*OsMADS18*), quality regulation (*RIN*, *OsMADS7*), and ideotype design (*ZmSOC1*, *BnaSVP*). These cases illustrate a “multi-effect single-target” strategy that concurrently optimizes metabolic flux, stress responses, and architectural traits.

The MADS-box gene family holds significant potential for molecular breeding owing to its multifaceted regulatory mechanisms. Regarding yield improvement, gene dosage analyses of *OsMADS18* (TM3/SOC1 subfamily) demonstrated that each additional copy induces an 8% elongation of basal leaf cells and intercalary elongation, increasing source leaf area and enhancing assimilate translocation to the panicle. Consequently, this results in an 8–12% yield increase while maintaining stable grain protein content (Figure 2) [69]. Also, building on the established role of *OsMADS26* in modulating drought responses, recent studies have successfully employed the CRISPR/Cas9 system to silence this gene in rice (*Oryza sativa*). This targeted knockout strategy significantly enhanced the resistance to drought tolerance and pathogens, demonstrating the potential of precise genome editing to optimize stress resilience without compromising yield [70]. Beyond yield traits, the capacity of MADS-box proteins to regulate quality metabolic networks at multiple nodes expands their utility in breeding programs. In tomato, the RIN (SEP subfamily) protein shares a SEP3-AG tetramer binding site with the promoter of the ethylene biosynthesis gene *E8*. Heterozygous mutations reduced *E8* expression by 35% and delayed ripening by 7–10 days. Concurrently, redirecting the glycolytic intermediate fructose-6-phosphate toward the ascorbic acid pathway increased soluble sugars by 12% and lycopene by 18%, while extending fruit firmness retention from 10 to 17 days without the defects associated with immature mutants (Figure 2) [71]. In rice, *OsMADS7* binds to the CArG box within the Waxy (Wx) promoter, sustaining granule-bound starch synthase I (GBSSI) activity under high-temperature stress. This maintains amylose content at 16–18%, reduces chalkiness by 40%, and mitigates the deterioration of commercial rice quality associated with warming climates (Figure 2) [32]. Collectively, these studies demonstrate that the MADS-box family offers a ‘multi-effect single-target’ strategy for enhancing crop quality by concurrently regulating metabolic flux allocation (e.g., sugar/starch synthesis) and stress responses (e.g., ethylene/heat signaling) (Figure 2) [32].

The MADS-box gene family also holds significant potential for crop ideotype design. In maize (*Zea mays*), *ZmSOC1* (TM3/SOC1 subfamily) exerts dual regulation via a shared CArG box in the *GA2ox4* promoter region: it reduces plant height by 12–18% while enhancing lodging resistance and optimizes photosynthetic nitrogen use efficiency to increase grain number per panicle by 9%, ultimately boosting yield by 12% and achieving synergistic improvements in both architecture and productivity (Figure 2) [72]. Constitutive overexpression of the endogenous SOC1 homolog *(ZmSOC1*) in transgenic lines accelerated flowering time and reduced plant height while maintaining individual grain yield [72]. These phenotypic modifications suggest that ZmSOC1 engineering facilitates high-density planting architectures, thereby unlocking greater yield potential per unit area through optimized population management [7]. In tetraploid rapeseed (*Brassica napus*), simultaneous knockout of *BnaSVP.A2* (SVP subfamily) and *BnaSVP.C2* (SVP subfamily) advances flowering by 5–7 days, avoiding heat-induced premature ripening while preserving oil content and fatty acid composition, thereby providing a framework for synchronous editing in polyploid crops (Figure 2) [73].

In summary, the breeding value of the MADS-box gene family is primarily realized through two mechanisms: incremental trait enhancement via fine-tuning of gene expression levels, and synergistic regulation of diverse pathways through signal integration. Collectively, these attributes establish the theoretical basis for an ‘expression level–phenotype’ quantitative relationship model, offering a novel, quantifiable, and verifiable approach for breeding design within multigenic networks. This field is undergoing a paradigm shift from conventional single-gene regulation to systematic molecular design breeding, facilitated by advanced technologies such as single-cell multi-omics. Future breeding strategies should leverage advanced technologies, including CRISPR/Cas9-based precision genome editing systems, single-cell multi-omics, and high-throughput functional validation. By pyramiding superior alleles of stress-resistance MADS-box genes and optimizing their expression patterns, it will be possible to develop novel crop varieties characterized by broad-spectrum stress resilience, yield stability, and superior quality. These innovations will ensure the stability of food and economic crop production, offering robust genetic solutions to mitigate the adverse impacts of global climate change.

## 5. Summary

A synthesis of MADS-box responses to diverse stresses reveals a highly conserved core mechanism: proteins function via CArG-box binding and dynamic complex formation through protein–protein interactions and post-translational modifications. Given their ancestral roles in development, these genes typically couple stress tolerance with developmental regulation. However, a critical subset possesses the unique capacity to decouple stress resistance from growth inhibition, thereby mitigating yield penalties and offering a pivotal advantage for crop improvement.

While the molecular basis is conserved, functional outcomes exhibit significant species-specific divergence driven by ecological niches and life-history strategies. Monocots (e.g., rice, wheat) prioritize yield stability via *SVP*/*SOC1*/*AGL17*-mediated networks, whereas dicots (e.g., Arabidopsis, tomato) employ more pathway-specific mechanisms. Furthermore, herbaceous crops rely on rapid hormonal responses, while woody perennials utilize dormancy-associated programs (e.g., *DAM* genes) for long-term adaptation. This diversity underscores the need to move beyond model systems to explore lineage-specific regulatory networks in non-model species.

Comparative genomic analyses demonstrate that the structural modularity and evolutionary divergence of MADS-box genes underpin functional conservation across monocot and dicot lineages, while lineage-specific differentiation drives agronomic trait plasticity. Recent empirical evidence underscores the pivotal roles of this family in yield determination (e.g., *OsMADS18*-mediated tillering in rice) [74], floral morphogenesis (e.g., regulatory innovations in Asteraceae) [75], fruit quality (e.g., *VvAGL12* (AGL12 subfamily) -associated growth in grapes) [76], and abiotic stress resilience (e.g., *SlMBP22* (AG subfamily)-conferred drought tolerance in tomato) [77]. However, critical knowledge gaps persist: the spatiotemporal dynamics of MADS-box protein interactions within hormone signaling networks remain incompletely elucidated; functional annotation of orthologs in non-model crops is insufficient, limiting breeding utility; and the precision of gene editing strategies regarding dosage and tissue specificity necessitates further optimization.

To address these challenges, the field is undergoing a paradigm shift from “single-gene, single-stress” studies toward multidimensional, network-oriented frameworks. First, cross-stress crosstalk is being elucidated to identify multifunctional targets (e.g., *OsMADS26*). Second, “growth-tolerance decoupling” is being engineered to break yield trade-offs. Third, multi-omics data is being integrated to construct comprehensive regulatory cascades involving chromatin remodeling. Fourth, resource mining is being expanded to wild germplasm and woody species. Concurrently, the integration of precise gene editing with agronomic practices is accelerating the translation of basic findings into resilient varieties.

This review synthesizes our current understanding of MADS-box transcription factor regulatory networks, highlighting that while significant progress has been made in model systems, a systematic framework for crop application remains elusive. Elucidating these mechanistic circuits offers substantial translational potential for engineering crops with enhanced performance and resilience. Ultimately, leveraging MADS-box gene functionality is imperative for advancing agricultural innovation and addressing global food security challenges.

## Data Availability

No new data were created or analyzed in this study.
